# Adapting an Intervention to Improve Acute Myocardial Infarction Care in Tanzania: Co-Design of the MIMIC Intervention

**DOI:** 10.5334/aogh.4361

**Published:** 2024-03-13

**Authors:** Julian T. Hertz, Kristen Stark, Francis M. Sakita, Jerome J. Mlangi, Godfrey L. Kweka, Sainikitha Prattipati, Frida Shayo, Vivian Kaboigora, Julius Mtui, Manji N. Isack, Esther M. Kindishe, Dotto J. Ngelengi, Alexander T. Limkakeng, Nathan M. Thielman, Gerald S. Bloomfield, Janet P. Bettger, Tumsifu G. Tarimo

**Affiliations:** 1 Duke Global Health Institute, Duke University, Durham, North Carolina, USA; 2 Department of Emergency Medicine, Duke University School of Medicine, Durham, North Carolina, USA; 3 Kilimanjaro Christian Medical Center, Moshi, Tanzania; 4 Kilimanjaro Christian Medical University, Moshi, Tanzania; 5 Department of Internal Medicine, Duke University, Durham, North Carolina, USA; 6 Duke Clinical Research Institute, Durham, North Carolina, USA

**Keywords:** Acute Myocardial Infarction, Co-design, sub-Saharan Africa, Implementation Science, quality improvement

## Abstract

**Background:**

Uptake of evidence-based care for acute myocardial infarction (AMI) is suboptimal in Tanzania, but there are currently no published interventions to improve AMI care in sub-Saharan Africa.

**Objectives:**

Co-design a quality improvement intervention for AMI care tailored to local contextual factors.

**Methods:**

An interdisciplinary design team consisting of 20 physicians, nurses, implementation scientists, and administrators met from June 2022 through August 2023. Half of the design team consisted of representatives from the target audience, emergency department physicians and nurses at a referral hospital in northern Tanzania. The design team reviewed multiple published quality improvement interventions focusing on ED-based AMI care. After selecting a multicomponent intervention to improve AMI care in Brazil (BRIDGE-ACS), the design team used the ADAPT-ITT framework to adapt the intervention to the local context.

**Findings:**

The design team audited current AMI care processes at the study hospital and reviewed qualitative data regarding barriers to care. Multiple adaptations were made to the original BRIDGE-ACS intervention to suit the local context, including re-designing the physician reminder system and adding patient educational materials. Additional feedback was sought from topical experts, including patients with AMI. Draft intervention materials were iteratively refined in response to feedback from experts and the design team. The finalized intervention, Multicomponent Intervention to Improve Myocardial Infarction Care in Tanzania (MIMIC), consisted of five core components: physician reminders, pocket cards, champions, provider training, and patient education.

**Conclusion:**

MIMIC is the first locally tailored intervention to improve AMI care in sub-Saharan Africa. Future studies will evaluate implementation outcomes and efficacy.

## Introduction

Recent studies have demonstrated that acute myocardial infarction (AMI) is common but frequently misdiagnosed in northern Tanzania [1,2,3]. Beyond misdiagnosis, uptake of evidence-based AMI care in the region is suboptimal: few patients presenting to the emergency department (ED) with possible AMI symptoms receive appropriate diagnostic testing for AMI, few patients with confirmed AMI are treated with appropriate medications in the ED, and few patients are taking evidence-based secondary preventatives following their hospitalization [1, 3,4,5]. This suboptimal care is associated with poor outcomes: the 30-day and 1-year mortality rate following AMI in northern Tanzania is among the highest in the world [3,4,5,6]. Qualitative and observational research from Tanzania and elsewhere in sub-Saharan Africa has identified multiple barriers to evidence-based AMI care, including lack of equipment, inadequate testing, limited training for providers, patient and provider awareness, and patient delays in care-seeking, among others [7,8,9,10,11]. These findings underscore the urgent need for strategies to improve the diagnosis and treatment of AMI in the region. However, interventions designed for the Tanzanian healthcare setting are lacking; a recent systematic review of quality improvement interventions for AMI care found no published studies from sub-Saharan Africa [12].

Evidence-based AMI care saves lives [13, 14], and extensive work has been done to improve uptake of such evidence-based care in high-income settings [12]. Various studies have found that hospital-based interventions in resource-replete settings can improve AMI diagnosis, treatment, and outcomes [15,16,17,18]. However, none of these published interventions are suitable for a hospital in Tanzania, a lower-middle income country where contextual factors differ substantially [7, 19, 20].

To address this critical gap, an interdisciplinary team sought to adapt an evidence-based approach to improving AMI care quality and outcomes in Tanzania. Guided by the ADAPT-ITT framework [21], the team incorporated iterative co-design methodology, which emphasizes the inclusion of the intervention target audience as partners in the design process [22, 23]. The co-design team integrated local contextual expertise, topical expertise, and research expertise. In this study, we report the adaptation of the first locally-tailored model to improve AMI care in Tanzania, the Multicomponent Intervention to Improve Myocardial Infarction Care (MIMIC). Although there are opportunities for improvement in multiple phases of AMI care in Tanzania [1, 4, 5, 7, 9,10,11, 19], this study focused on improving the proper diagnosis and treatment of AMI in the ED setting. The methodology and findings from this study may inform adoption elsewhere in sub-Saharan Africa, where contextual factors for AMI care are likely similar.

## Methods

### Setting

This study was conducted at Kilimanjaro Christian Medical Centre (KCMC), a tertiary referral hospital in northern Tanzania that serves a population of approximately 15 million people. KCMC was the site of our team’s formative research, which revealed opportunities for improvement in AMI treatment and identified barriers to care [1, 3,4,5,6,7]. KCMC is equipped with electrocardiography, troponin assays, echocardiography, and basic AMI therapies, including antiplatelet agents, anticoagulants such as heparin, and thrombolytics. Currently, KCMC does not have capacity for advanced cardiac procedures such as percutaneous coronary intervention or coronary artery bypass surgery; the nearest facility capable of performing percutaneous coronary intervention is in Dar es Salaam, approximately 10 hours away by road. The KCMC ED is staffed by a mix of physicians, residents, interns, clinical officers, and nurses; there are currently no formally trained cardiologists on staff at KCMC.

### Adapt-ITT

Although no *a priori* assumptions were made about what components would be included in the intervention, we ultimately decided to adapt an intervention from another setting [24] and adapt it to the Tanzanian context. The ADAPT-ITT model (Assessment, Decisions, Adaptation, Production, Topical experts, Integration, Training staff, and Testing) was used to guide the adaptation process [21]. The ADAPT-ITT model was first developed for adapting evidence-based HIV interventions but has been successfully utilized for adaptation of multiple types of interventions, including violence prevention programs, health education programs, mental health programs, and caregiver support programs [25, 26]. The original ADAPT-ITT model recommended eliciting feedback from the target population in a few of the adaptation steps. Instead of consulting our target population, providers in the KCMC ED, we used a co-design approach with KCMC providers to incorporate their critical feedback throughout the entire design and adaptation process.

### Co-design

The co-design methodology emphasizes partnership with the target audience at every stage of intervention development [22, 23]. Co-design principles, including active participation of the target population, are particularly important when adapting an intervention to a new setting and when some members of the investigator team are not members of the target community. For this study, the primary target population was KCMC ED providers, and so key members of the ED staff were invited to participate in the design process, as described below. KCMC providers functioned as full, equal partners throughout the design process, and they also served as experts on local context. Although patients were not the primary target of the intervention, the intervention ultimately included some patient-facing elements. For this reason, patients were also invited to participate in the design process for these elements, as described below.

### Design Team

The design team for the intervention consisted of physicians and nurses, cardiologists, implementation scientists, social scientists, departmental administrators, local health officials, patients, and other local stakeholders. Design team members were selected by the investigator team (which included the department head of the KCMC ED) by virtue of their expertise in the local context, implementation science, or intervention development. The design team included participants based in Tanzania and participants based primarily in the United States; all participants in the United States had prior experience conducting research in Tanzania and were familiar with the local context. Because the intervention was primarily focused on physician and nurse behaviors, patients did not participate in the majority of the design team sessions. However, patients were invited to participate in the design of patient-facing components of the intervention, as described below. The design team consisted of 20 members, as summarized in Table 1. In addition to the 20 standing members of the team, members of the KCMC ED staff were invited to participate in design team meetings on a rotating basis to include a multitude of perspectives from those whom the intervention would ultimately target.

**Table 1:** Characteristics of design team members (n = 20).


Primary occupation	n (%)

ED nurse	3 (15%)

ED Physician	3 (15%)

Implementation scientist	3 (15%)

Departmental administrator	2 (10%)

Internal medicine physician	2 (10%)

Clinical officer	2 (10%)

Master’s student	2 (10%)

Cardiologist	1 (5%)

Social scientist	1 (5%)

Ministry of health representative	1 (5%)

Nationality	

Tanzania	12 (60%)

United States	8 (40%)

Gender	

Male	12 (60%)

Female	8 (40%)


### Adaptation Process

The intervention was developed iteratively through a series of design team meetings, which occurred approximately once per month over a fifteen-month period (June 2022–August 2023). Design team meetings occurred in-person at the study hospital, but a hybrid format was used to allow some team members to participate virtually via teleconference. Design team meetings typically lasted for 1–2 hours, and design team members were provided refreshments and monetary compensation for travel (5000 Tanzanian shillings, approximately 2 USD). Meetings began with a summary of progress, followed by an overview of the meeting goals, a presentation of any additional data, and an open discussion. A total of 10 design team meetings were held, but substantial intervention and development work occurred between meetings. Examples of tasks that design team members performed between meetings included: conducting literature searches of effective interventions, meeting with the hospital medical records team to assess the feasibility of building intervention components within the electronic medical record system, meeting with patients to get their feedback on educational materials, printing draft intervention materials, translating materials into Swahili, and conducting interim audits of ongoing care to inform intervention development.

### Data Collection and Analysis

#### Assessment

Step one of the ADAPT-ITT framework, Assessment, calls for interviews and a needs assessment with the target population. We chose a mixed-methods approach using four different data sources for our needs assessment. First, the design team reviewed the results of recent published studies from KCMC demonstrating sub-optimal use of electrocardiograms and troponin testing, infrequent aspirin administration, and high 30-day and 1-year mortality [1, 3,4,5]. Secondly, because these published studies were conducted in 2019, the design team decided to obtain more recent data by conducting an audit. To do so, the design team reviewed 75 consecutive ED patients who met objective electrocardiographic and cardiac biomarker criteria [27] for AMI in 2022 from an ongoing AMI surveillance study. Thirdly, the design team reviewed the results of a survey of KCMC ED providers and nurses. This survey, which is the subject of a separate pending publication, was conducted to assess provider attitudes towards a potential quality improvement program for AMI care. Finally, in addition to this quantitative data, the design team reviewed the results of two formative studies regarding AMI care in Tanzania: a qualitative study of physician-perceived barriers to emergency care [7] and a comprehensive assessment of barriers and facilitators to evidence-based AMI care guided by the Consolidated Framework for Implementation Research (CFIR) [28] conducted among patients, providers, and healthcare administrators (publication pending).

#### Decision

The second phase of the ADAPT-ITT model involves reviewing existing interventions or programs to either adopt or adapt to a new context. To identify potential strategies for adaptation, the team reviewed all of the studies included in a recent systematic review of quality improvement interventions for AMI care [12]. The team supplemented this list of interventions with their own literature review of PubMed [using the search teams “quality improvement” AND (“myocardial infarction” OR “acute coronary syndrome”)]. The list of potential interventions reviewed by the team employed a range of strategies, including focusing on patient education, utilization of cultural and religious leaders, provider training, audit/feedback, and multicomponent interventions [12, 24, 29,30,31,32,33,34,35,36,37]. After reviewing these interventions, the design team selected an intervention for adaptation to the Tanzanian setting. Principle considerations in the selection of an intervention for adaptation were focus on improving both diagnosis and treatment, demonstrated efficacy, congruity with previously identified barriers in Tanzania, and feasibility within the local context.

#### Adaptation

Over the course of a one-year period (August 2022–July 2023), the design team met approximately once per month to iteratively adapt the selected intervention to the Tanzanian process. Following each meeting, detailed meeting notes were sent to the entire team to update members unable to participate synchronously and allow for their feedback. Any asynchronous feedback was presented at the start of the subsequent meeting. Two members of the design team (KS and JTH) took primary responsibility for analyzing and summarizing the results of in-person and asynchronous feedback from each meeting and presenting it at the following meeting. Substantial work was done by members of the design team between meetings, as described above.

#### Production, Topical Experts, and Integration

Draft materials for all intervention components were created by Tanzanian members of the team with extensive experience working at the study hospital (TGT and FMS). These draft materials were shared with the full design team, as well as topical experts, patients, and key informants from the ED staff. The process of production, feedback from topical experts, and integration occurred iteratively over several months (March 2023–July 2023) as intervention materials were further refined. Although topical experts were included throughout the co-design and adaptation process as members of the design team, additional input was sought from external topical experts, who were selected based on their expertise in implementation science, cardiology, and KCMC ED care processes. Furthermore, a member of the design team met individually with patients (n = 5) who had recently been admitted to the study hospital with AMI. These patients, selected via convenience sampling, were asked to review the patient-facing component(s) of the intervention and give feedback about comprehensibility and design.

#### Integration

In an iterative cycle, feedback from topical experts and key informants was integrated into the intervention, and materials were further refined.

#### Training and Testing

After finalizing the adapted intervention, all KCMC ED staff members were trained by members of the design team in the delivery of the intervention through a series of staff meetings in August 2023. The adapted intervention was implemented in the KCMC ED beginning on September 1, 2023, as part of a pilot implementation study. The results of this pilot trial will be reported separately.

## Ethics

This study received ethical approval from institutional review boards at Duke Health (Pro00090902), KCMC (Proposal No. 893), and the Tanzania National Institute of Medical Research (NIMR/HQ/R.8/Vol IX/2436).

## Results

### Phase I – Assessment

The design team reviewed the results of recent studies from KCMC describing AMI care processes in 2019 [1, 3,4,5]. Key findings from these studies were: less than 3% of ED patients with chest pain or dyspnea received both an electrocardiogram and cardiac biomarker testing, only 23% of ED patients with AMI were treated with aspirin, 43% of AMI patients died within 30 days of initial hospital presentation, and the one-year mortality rate following AMI was 60%.

The results of the random audit of 75 recent AMI cases are presented in Table 2. The audit revealed that most of these patients did not have a documented diagnosis of AMI despite meeting objective electrocardiographic and cardiac biomarker criteria, few were treated with aspirin, and many died within 30 days (Table 2).

**Table 2:** Results of audit of care processes among AMI patients in the KCMC ED, 2022 (N = 75).


CARE PROCESS	n	(%)

Documented diagnosis of AMI	12	(16)

Treated with aspirin	8	(11)

Survived to 30 days	47	(67)


The design team reviewed the results of two recent qualitative studies exploring barriers to emergency AMI care at KCMC [7]. The design team reviewed these findings and identified the most prominent barriers that could be addressed with an ED-based intervention (Table 3).

**Table 3:** Primary barriers to evidence-based AMI care in the KCMC ED identified from prior qualitative work [7] (publication pending).


BARRIER	INFORMANT

Patients have limited understanding of AMI	Patients, providers, administrators

Providers fail to consider the diagnosis of AMI	Providers, administrators

Providers have insufficient training in AMI care	Providers, administrators, patients

Lack of designated leader(s) for improvement efforts	Providers, administrators


Furthermore, the design team reviewed the results of an as yet unpublished survey of attitudes towards MI quality improvement among KCMC staff performed by this study team. Key findings of this survey included: (a) 100% of providers agreed that a quality improvement intervention was needed to target AMI care at KCMC; (b) 100% agreed that they would be interested in participating in a quality improvement program; and (c) when asked to identify which single strategy would be most effective in improving AMI care, the most commonly selected strategies were provider training and patient education.

After reviewing all of the above data, the design team agreed unanimously that a quality improvement intervention was needed to improve AMI care at KCMC.

### Phase II – Decision

The design team reviewed a variety of published quality improvement interventions that employed a range of strategies [12, 24, 29,30,31,32,33,34,35,36,37]. After review of the needs assessment data and further discussion, the design team determined that a multifaceted intervention should be adapted, with a focus on provider training and awareness. Having considered multiple options, the team chose to adapt the BRIDGE-ACS (Brazilian Intervention to Increase Evidence Usage in Acute Coronary Syndromes) intervention [24], due to its multicomponent nature, its focus on ED-based care, its use in a resource-limited setting, its focus on both diagnosis and treatment, and its efficacy. Furthermore, BRIDGE-ACS seemed feasible for the KCMC setting because it did not require expensive technology or additional staff. Finally, the BRIDGE-ACS intervention emphasized training providers and reminding them to consider the diagnosis of AMI—two strategies that were well-suited to the barriers to AMI care previously identified in Tanzania [7].

### Phase III – Adaptation

The design team spent several months discussing appropriate modifications to the BRIDGE-ACS study. The original multi-component intervention consisted of provider reminders, a checklist, case management, and educational materials for providers.

#### Provider Reminders

The BRIDGE-ACS utilized two forms of provider reminders: a bright yellow “Chest Pain” sticker attached to the clinical evaluation form as a rapid triage tool and color-coded patient wristbands to identify patients with different forms of AMI. These strategies could not fit within the workflow of the KCMC ED, where patient wristbands and paper triage tools are not used. To easily integrate the physician reminders into the existing workflow, the design team opted to utilize the existing ED triage card system. In this system, the triage nurse attaches a color-coded card to the patient’s stretcher to indicate the patient’s stage of care (red for not yet seen by a provider, yellow for in progress, green for ready for disposition). The design team decided to add an additional type of triage card for patients with possible AMI symptoms that would be attached to the patient stretcher to remind physicians that this patient may have AMI. The team reasoned that a red card stating “Possible AMI Patient” would prompt the physician to consider this diagnosis.

#### Checklist

In the BRIDGE-ACS intervention, after a patient with potential AMI is identified, the ED physician is given a checklist for orders containing an algorithm for testing, treatment, and risk stratification. This strategy required adaptation for our setting, because the KCMC ED does not use a paper ordering system, and order sets are currently not possible within the hospital’s electronic medical record system. Therefore, the design team decided to modify the checklist into a laminated pocket card given to all ED providers that would review AMI diagnosis and treatment.

#### Educational Materials for Providers

The package of educational materials in BRIDGE-ACS consisted of a website with presentations about AMI and posters displaying guidelines for the management of AMI. Posters outlining AMI management guidelines are already on display in the study hospital, so this component of BRIDGE-ACS did not need to be added in our setting. The design team created a brief (approximately 30-minute) online training module for new ED providers that presents a brief overview of AMI diagnosis and treatment and also includes questions to assess knowledge. If any questions are answered incorrectly, the quiz redirects the respondent to more detailed information regarding AMI diagnosis and treatment.

#### Case Management

The BRIDGE-ACS intervention employed a trained nurse as a case manager who was responsible for following up with AMI patients, ensuring the intervention was utilized for each patient, and identifying barriers to implementation. The design team agreed to keep this role but opted to reframe it as a ‘champion’ for our intervention. During discussion on the champion role, it became evident it would be too much work for a single provider. The team decided to create a physician champion and a nurse champion role, each with distinct yet overlapping responsibilities. Both would be responsible for following up on individual AMI cases and ensuring the successful implementation of the overall intervention. The physician champion would be responsible for ensuring the physicians complete the training module and receive the pocket cards and would also have the responsibility for addressing physician-related barriers to implementation. The nurse champion would be responsible for ensuring that the nurses complete the training module and use the AMI triage cards correctly, and would also be responsible for addressing any nurse-related barriers to implementation.

#### Educational Materials for Patients

Although patient education was not a core component of the BRIDGE-ACS intervention, the design team agreed that there was a critical need to improve patient education regarding AMI. Therefore, the team elected to create an educational pamphlet to be given to patients with AMI while they are in the ED. More comprehensive educational interventions for patients in the inpatient and follow-up settings were deferred to future quality improvement studies.

### Phase IV – Production

Draft materials for all intervention components (triage reminder cards, pocket cards, online training modules, champion responsibilities, patient pamphlets) were created and shared with the full design team, topical experts, patients, and key informants from the ED staff, as described below.

### Phase V – Topical Experts

Additional input was sought from external implementation scientists, cardiologists, KCMC ED staff who had not participated in the design process, and five patients who had recently been admitted to KCMC with AMI. Topical experts were asked to review the draft intervention materials and give feedback about comprehensibility, appropriateness, and design. Patient topical experts were only asked to give feedback about the patient educational pamphlet. Table 4 summarizes the refinements to the intervention suggested by topical experts.

**Table 4:** Topical expert recommendations for refinements to a quality improvement intervention for AMI care in Tanzania.


INTERVENTION COMPONENT	REFINEMENT(S)	TOPICAL EXPERT(S)

Triage reminder cards	(1) Affix to a cable for easy attachment to stretchers; (2) Post list of AMI symptoms in the triage area to aid nurses in recognition of possible AMI cases	ED nurses

Pocket cards	Minor refinements to wording	Cardiologists, ED physicians

Online training modules	Minor refinements to wording	Cardiologists, implementation scientists

Champions	(1) Create certificates of appreciation for staff who provide excellent care; (2) Assign responsibility for ensuring adequate supply of aspirin in the ED to the nurse champion	ED nurses, ED physicians, implementation scientists

Patient educational pamphlets	(1) Increase font and add color; (2) Minor refinements to wording; (3) Project digital versions ofpamphlets on the screens in the waiting room	Patients, ED nurses, ED physicians


### Phase VI – Integration

In an iterative cycle, feedback from topical experts and key informants was integrated into the intervention, and materials were further refined. Refinements that occurred in this stage included: (1) adding a laminated poster listing common AMI symptoms in the triage area to help triage nurses identify appropriate patients for the AMI triage reminder cards; (2) assigning the physician champion the additional responsibility of recognizing staff who provide exemplary AMI care with certificates of appreciation; (3) assigning the nurse champion the additional responsibility of ensuring an adequate supply of aspirin in the ED medicine cabinet on a daily basis; and (4) projecting digital versions of the patient educational pamphlet onto the television screens in the ED waiting room (Table 4).

### The MIMIC Intervention

**Figure 1:**
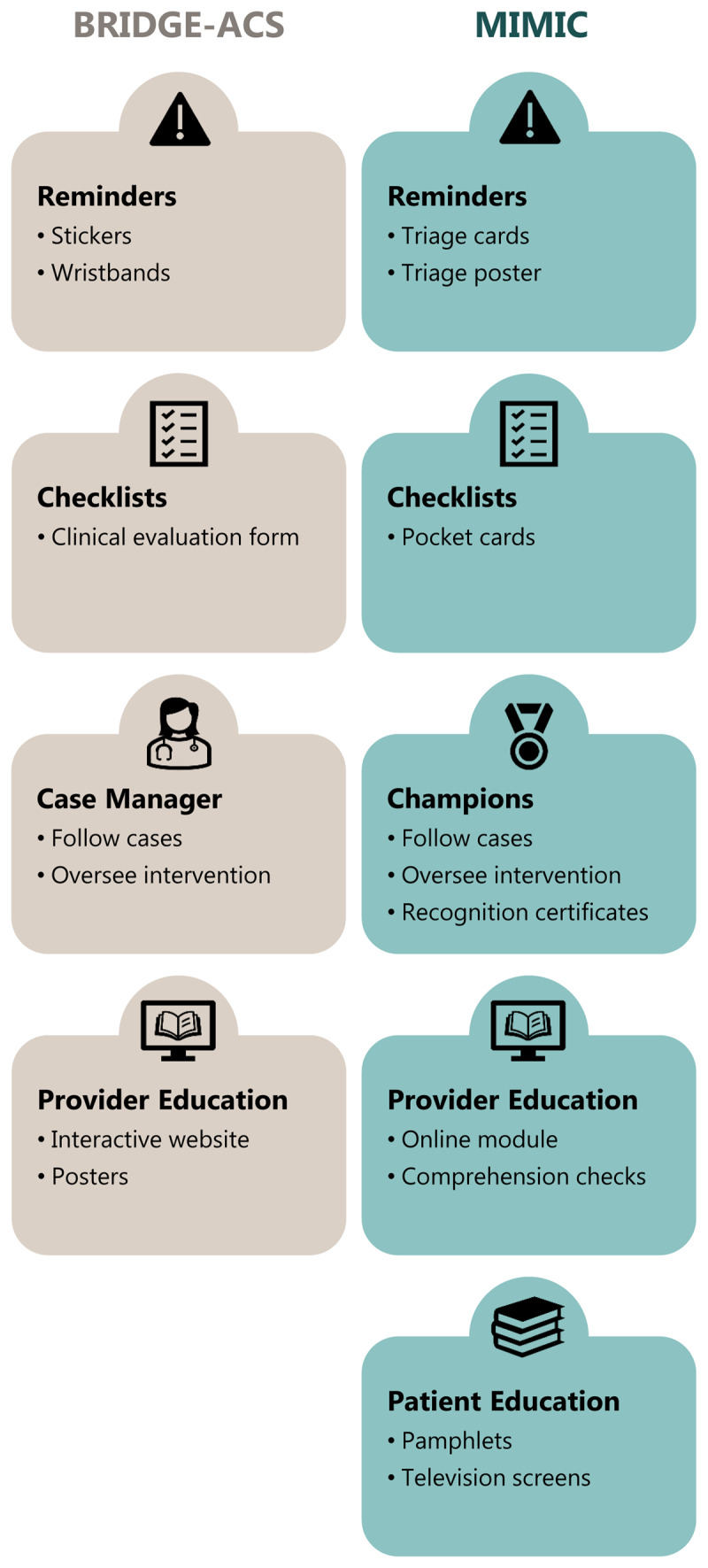
Adaptation of the BRIDGE-ACS intervention to the MIMIC intervention to improve AMI care in Tanzania.

The finalized components of the adapted intervention, the Multicomponent Intervention to improve Myocardial Infarction Care in Tanzania (MIMIC), are presented in the Supplementary Material. Figure 1 summarizes the core components of the BRIDGE-ACS intervention and their adaptation into the MIMIC intervention.

**Figure 2:**
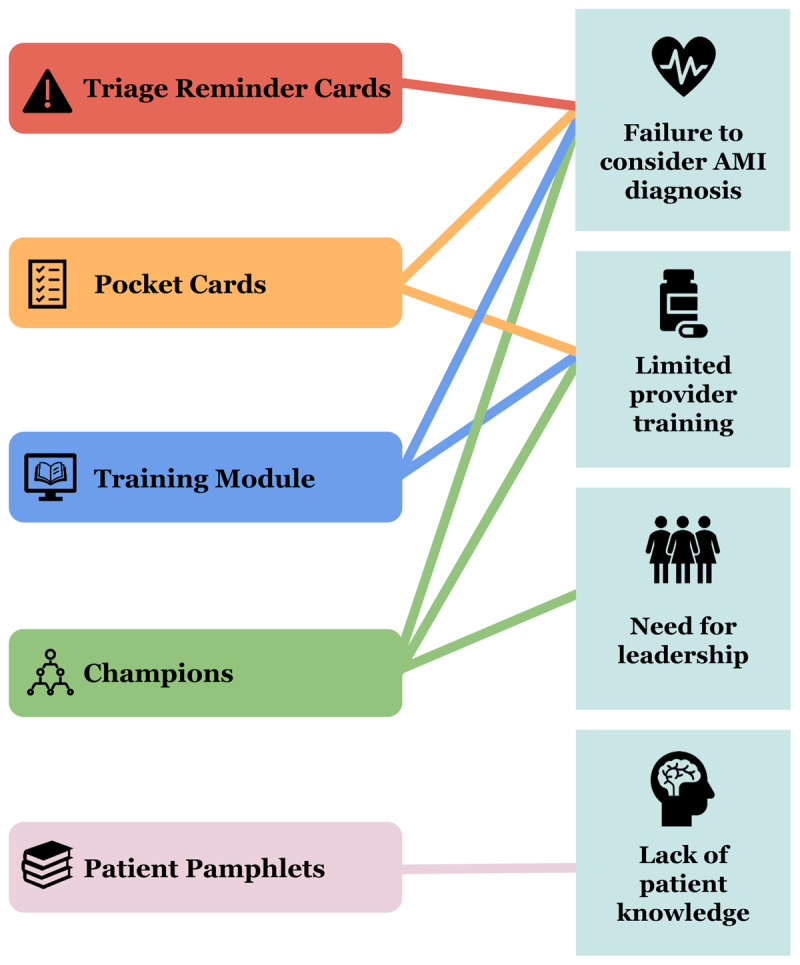
Mapping the MIMIC intervention to the barriers targeted by each intervention component.

Figure 2 summarizes the primary barriers to AMI care identified by Design Team (see Table 3) and the components of the MIMIC intervention that target each barrier.

### Phases VII and VIII – Training and Testing

**Figure 3:**
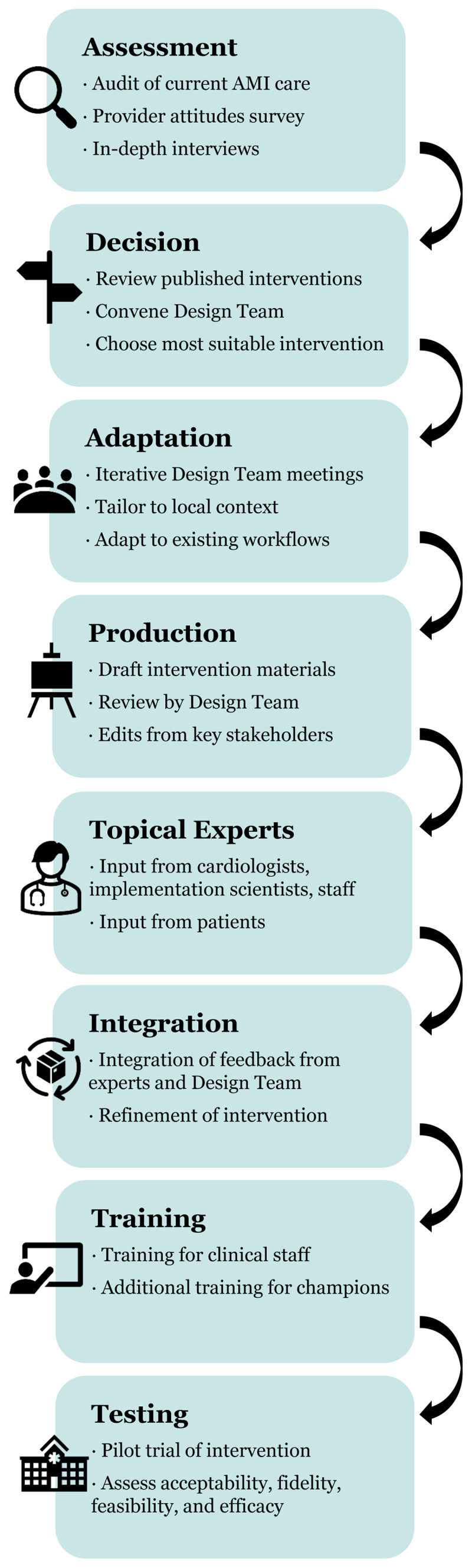
Use of the ADAPT-ITT framework to develop a quality improvement intervention for myocardial infarction care in Tanzania.

The KCMC ED staff was trained in all MIMIC intervention components through a series of three staff meetings in August 2023. Additional meetings were held with the designated nurse and physician champions to train them about their responsibilities. Testing of the adapted intervention is ongoing. A pilot trial is currently being conducted in the KCMC ED to assess the acceptability, feasibility, fidelity, and effect of MIMIC on AMI care processes. The results of this trial will be published separately. Figure 3 summarizes the use of the ADAPT-ITT framework in this study, and Table 5 summarizes key findings from each step of the adaptation process.

**Table 5:** Summary of key findings from adaptation of an intervention to improve AMI care in Tanzania using the ADAPT-ITT framework.


ADAPT-ITT STEP	OUR APPROACH	KEY FINDINGS

1. Assessment	(a) Review previously published data describing AMI care at KCMC, (b) Audit ED care for 75 recent AMI cases, (c) Review results of provider attitudes survey, (d) Qualitative data from in-depth interviews with providers, patients, and administrators.	(a) Diagnostic workups for AMI are not routine at KCMC; (b) Most patients with AMI do not receive aspirin in the ED; (c) Providers are strongly interested in a quality improvement intervention; (d) Provider training and patient education are barriers to care.

2. Decision	(a) Review systematic review of AMI quality improvement studies, (b) Conduct literature search of quality improvements strategies for AMI, (c) Convene Design Team to select an intervention that is appropriate for the local context.	Selection of the BRIDGE-ACS for adaptation to the Tanzanian context.

3. Adaptation	Iterative Design Team meetings to review components of the original intervention and tailor them to the local context.	(a) Use of triage cards instead of patient wristbands; (b) transforming checklists to pocket cards; (c) use of champions instead of case managers; (d) addition of a patient educational pamphlet.

4. Production	Production of draft materials for Design Team and topical expert review. Production, Topical Experts, and Integration occurred iteratively.	Minor refinements to wording of training module, pocket card, and educational pamphlet.

5. Topical Experts	Input from implementation scientists, cardiologists, KCMC staff, and 5 recent AMI patients.	(a) Additional champion responsibilities created, including awarding congratulatory certificates and ensuring adequate supply of aspirin; (b) Electronic displays of educational pamphlet in the ED waiting room; (c) Additional minor refinements to educational pamphlet

6. Integration	Iteratively integrated feedback from topical experts and the design team to further refine all intervention components.	Finalization of the MIMIC intervention

7. Training	ED staff were trained by members of the Design Team during three staff-wide meetings. Additional training meetings held with designated champions.	Training completed in August 2023

8. Testing	Pilot trial of the MIMIC intervention to be conducted in the KCMC ED to assess acceptability, fidelity, and preliminary effect on care processes.	Results pending


## Discussion

To our knowledge, this paper presents the first systematically-derived, locally tailored intervention to improve AMI care in sub-Saharan Africa. As the burden of noncommunicable disease continues to rise in the region [38], there is a growing need for contextually appropriate quality improvement interventions for AMI and other cardiovascular diseases. In the present study, we present the process of co-design and adaptation of a quality improvement intervention for ED-based AMI care in northern Tanzania. Future studies will evaluate implementation outcomes and the efficacy of the intervention.

Attention to local contextual factors is essential when adapting an intervention to a new setting [28], and the process of developing the MIMIC intervention underscored this point. Nearly all components of the original BRIDGE-ACS intervention [24] required substantial modification for adaptation in our setting. For example, the original intervention used patient wristbands and paper evaluation forms, which were not suitable for our setting as these items were not part of the existing workflow. Tailoring an intervention to the local context contributes to maximal impact [39], although the efficacy of the MIMIC intervention is currently unknown. In Brazil, the BRIDGE-ACS intervention resulted in a significant increase in the use of evidence-based AMI therapies but did not have a significant effect on 30-day mortality [24]. In northern Tanzania, where current uptake of evidence-based therapy is lower and 30-day mortality is higher than in the BRIDGE-ACS control hospitals [3, 5, 24], a well-designed quality improvement intervention may have a greater impact on care processes and outcomes. Our future work will test this hypothesis. The MIMIC intervention developed in this study included several strategies targeting provider and patient knowledge, which were important barriers to care identified in prior qualitative work [7]. Future work will need to evaluate whether these components of the intervention have an impact on provider and patient understanding, in addition to care processes and clinical outcomes.

As the burden of AMI in sub-Saharan Africa continues to rise [40], there will be a growing need for research programs to monitor AMI care and implement interventions to maximize uptake of evidence-based care. To date, only a handful of published studies describe AMI care patterns in the region, and these studies have mostly been conducted at specialized cardiac centers with access to cardiologists and advanced coronary interventions [41,42,43]. Unfortunately, access to such advanced cardiac care is currently extremely limited in sub-Saharan Africa [44, 45], and so the vast majority of AMI care is likely to occur in hospital settings like ours in the near future. Thus, there is a critical need for feasible and locally relevant quality improvement interventions for AMI care in resource-limited hospitals in sub-Saharan Africa. To our knowledge, the MIMIC intervention presented here is the first such intervention published from the region; the strategies and methodology presented here can serve as a model for teams working to improve AMI care at similar hospitals across sub-Saharan Africa.

This study had several strengths, including using a co-design approach, which allowed the target audience to participate as equal partners in every step of the design and adaptation process. Co-design allows for the development of interventions that are more acceptable, appropriate, and sustainable in the local context [22, 23]. Moreover, using the evidence-based ADAPT-ITT framework to guide our adaptation process strengthened the rigor and reproducibility of our work [21]. Alimitation of this study was its focus on a single hospital, which allowed for extensive tailoring to a specific context but limited generalizability to other settings. Further adaptations and contextual tailoring will likely be required prior to implementing the MIMIC intervention in other settings in sub-Saharan Africa. Although this intervention was developed for a single center, our prior qualitative work conducted at multiple healthcare facilities found that contextual factors influencing AMI care are similar across Tanzania [7]. Given the absence of other published quality improvement interventions for AMI care in sub-Saharan Africa, MIMIC could serve as an excellent starting point for hospitals seeking to implement their own quality improvement initiatives elsewhere in sub-Saharan Africa. Additional adaptation to tailor the intervention to the specific hospital would be necessary, and the co-design methodology guided by the ADAPT-ITT framework that we present here could serve as a roadmap for the local adaptation process. Importantly, the MIMIC intervention was developed to target the ED phase of AMI care; additional work is needed to develop contextually appropriate interventions for other phases of AMI care, such as prevention and maintenance.

In conclusion, this study presents the first systematically-derived, locally-tailored intervention to improve AMI care in Tanzania, MIMIC. Additional study is needed to develop and test contextually adapted quality improvement interventions for AMI across sub-Saharan Africa.

## Data Accessibility Statement

All data for this study is contained in the manuscript and supplementary material.

## Additional File

The additional file for this article can be found as follows:

10.5334/aogh.4361.s1 Supplementary Material. The MIMIC Intervention: Multicomponent Intervention to Improve Myocardial Infarction Care in Tanzania.
