# Supplementary material for: Adapting an Intervention to Improve Acute Myocardial Infarction Care in Tanzania: Co-Design of the MIMIC Intervention
Source: Ann Glob Health. 2024 Mar 13;90(1):21. doi: 10.5334/aogh.4361 (PMC10941691; doi:10.5334/aogh.4361)
Supplement: Supplementary Material. — The MIMIC Intervention: Multicomponent Intervention to Improve Myocardial Infarction Care in Tanzania. [file agh-90-1-4361-s1.pdf]

The MIMIC Intervention

Multicomponent Intervention to Improve Myocardial Infarction Care in Tanzania

This intervention was designed to improve care of acute myocardial infarction (AMI) in the Emergency Department (ED) at Kilimanjaro Christian Medical Center in Moshi, Tanzania. The intervention consists of 5 components:

1. Triage reminder cards
2. Online refresher module
3. Pocket cards
4. Champions
5. Patient educational pamphlets

These components are described in further detail on the following pages. For additional questions about the MIMIC intervention, please contact the MIMIC study PI, Dr. Julian Hertz at julian.hertz@duke.edu

1. Triage Reminder Cards

Triage nurses place a special red triage card on the stretchers of all new patients presenting to the ED with possible AMI symptoms. To help the triage nurses remember these symptoms, a large sign is posted by the triage desk with AMI symptoms listed.

Triage poster:

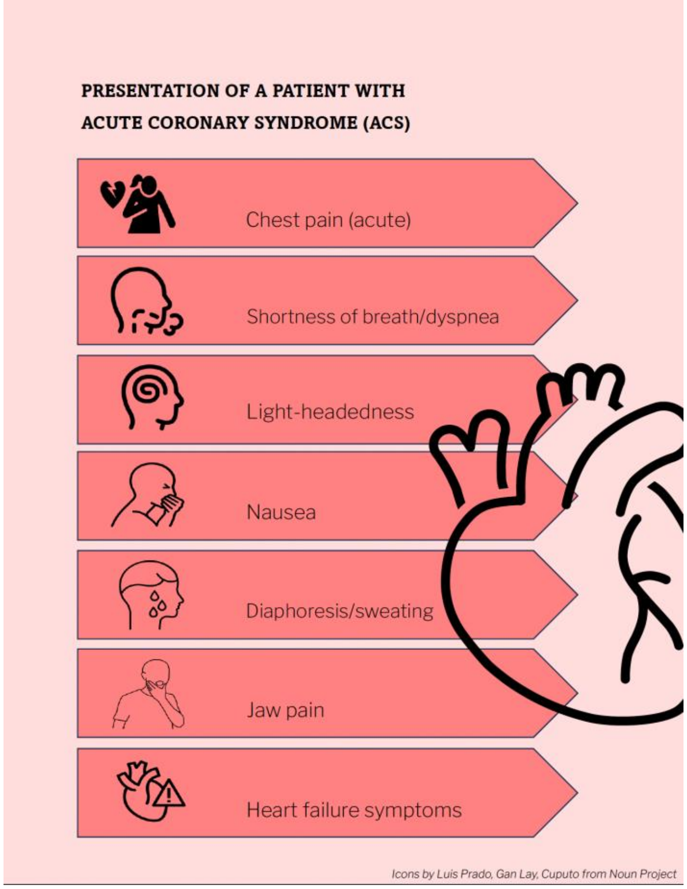

A picture of the red triage card used in MIMIC is below:

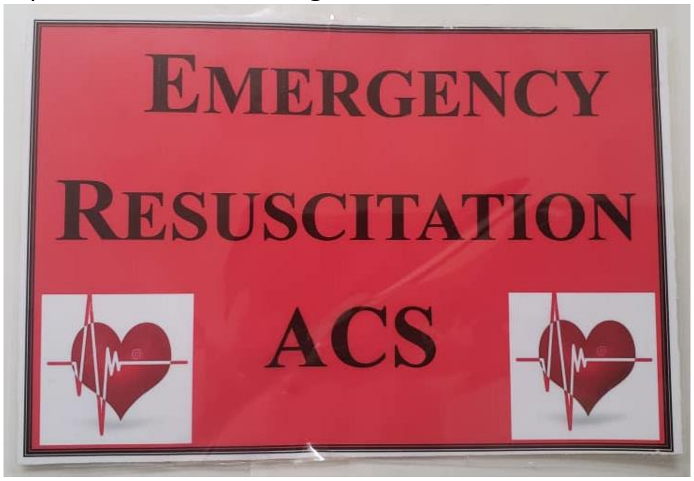

2. Online refresher module

All providers (doctors, nurses, clinical officers) working in the ED are required to complete an online training module that reviews AMI diagnosis and treatment in the ED setting. This module was designed using the Google Forms platform. You can directly access the online module using this link:

https://docs.google.com/forms/d/e/1FAIpQLSfyeHq8nKBhJFB9gBeotU3QIX6cqeUV5qeRzVaN9N1EGdRj3A/viewform?usp=sf_link

The link to the module was shared with ED staff by the Champions (described below). The Champions were responsible for monitoring the module to ensure all staff had completed it and for sending the link to new and rotating staff.

Below is a copy of the full module:

Acute Coronary Syndrome
Refresher Module for Emergency Medicine Providers

Pathophysiology

Acute Coronary Syndrome (ACS) is a group of diseases that occur when there is decreased oxygen delivery to cardiac muscle, resulting in ischemia or infarction of cardiac cells (cardiomyocytes). This most commonly occurs due to partial or complete blockages in the coronary arteries. There are many risk factors for ACS. Some of the most common are older age, hypertension, diabetes mellitus, hypercholesterolemia, obesity, tobacco use, and HIV. *However, even patients with no known risk factors can develop ACS—especially patients with undiagnosed comorbidities.*

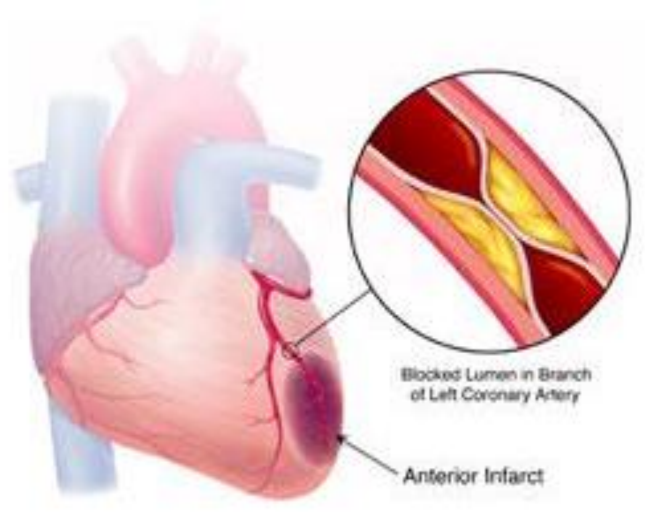

Presentation

ACS can present with a wide range of symptoms. The most common symptoms of ACS are:

- -Chest pain
- -Shortness of breath/dyspnea
- -Light-headedness/dizziness
- -Nausea
- -Jaw pain
- -Diaphoresis/sweating

There are other symptoms of ACS that are less common, such as epigastric pain, shoulder pain, and syncope. *Importantly, approximately 10% of patients with ACS will present with atypical symptoms; therefore it is important to maintain a high index of suspicion for ACS, even in patients without chest pain.*

<u>Question 1:</u>

Which of the following patients presenting to the Emergency Department should undergo testing for ACS?

a) A 35 year-old man with history of diabetes who presents with one week of epigastric pain radiating to the shoulder, nausea, and shortness of breath
b) A 52 year-old woman with no known medical history who presents with several days of worsening chest pressure and diaphoresis
c) A 74 year-old woman with history of hypertension and stroke who presents with one day of jaw pain, light-headedness, and dyspnea on exertion.
d) All of the above

Correct Answer: That is correct! All of these presentations are consistent with ACS, so testing for ACS should be done for all of these patients.

Incorrect Answer: That is incorrect. The correct answer is “d) All of the above.” All of these presentations are consistent with ACS, so testing for ACS should be done for all of these patients.

<u>Definitions</u>

ACS consists of two diagnoses: myocardial infarction and unstable angina.

Myocardial infarction describes a case where there has been complete death (infarction) of some cardiac muscle (cardiomyocytes). There are two types of myocardial infarction: ST Elevation Myocardial Infarction (STEMI) and non-ST Elevation Myocardial Infarction (NSTEMI). A STEMI is defined by ACS cases where specific electrocardiogram (ECG) features are seen, as discussed later. Most STEMI cases involve a complete occlusion of a coronary artery and transmural (all the way through the wall) infarct of the heart. NSTEMI is defined by an abnormally elevated troponin (or other cardiac biomarker) that is rising or falling due to infarcted heart muscle. Many (but not all) NSTEMI cases are caused by partial blockages of coronary arteries that involve non-transmural infarcts of the heart.

Unstable angina describes ACS cases where the ECG does not demonstrate a STEMI and the troponin test is normal (ie, negative, or below the recommended cutoff value). This occurs in cases where there are partial blockages of coronary arteries that are causing ischemia of cardiac muscle without infarction. Ischemia is a state where the cardiac muscle cells are temporarily and reversibly dysfunctional. Unstable angina is diagnosed clinically based on the presentation of worsening ACS symptoms, such as exertional chest pain that is worsening or now occurring at rest. Unstable angina symptoms can be intermittent.

Importantly, the approach to diagnosis and initial treatment of all forms of ACS (STEMI, NSTEMI, unstable angina) is similar.

STEMI Diagnosis

A STEMI is defined by ≥1 mm of ST elevation in at least 2 contiguous leads. The ST segment is the segment between the QRS complex and the T wave:

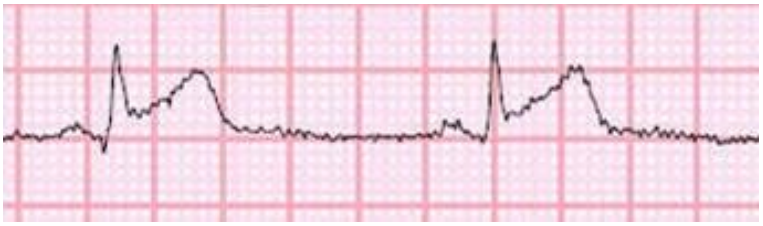

“Contiguous leads” refer to ECG leads that are geographically adjacent in the heart. The following groups of ECG leads are contiguous:

-Lateral leads: I, aVL
-Inferior leads: II, III, aVF
-Anterior leads: V1-V6

Therefore, a patient with ≥1 mm of ST elevation in leads I and aVL would meet criteria for a STEMI because these leads are contiguous. Similarly, a patient with ≥1 mm of ST elevation in leads III and aVF would also meet criteria for a STEMI. Of note, the threshold for a STEMI is slightly different in leads V2 and V3. In these leads, the threshold for a STEMI is ≥1.5 mm for women, ≥2mm for men aged 40 years or greater, and ≥2.5 mm for men younger than 40 years old. Here are some examples of STEMI ECGs:

STEMI Example #1:

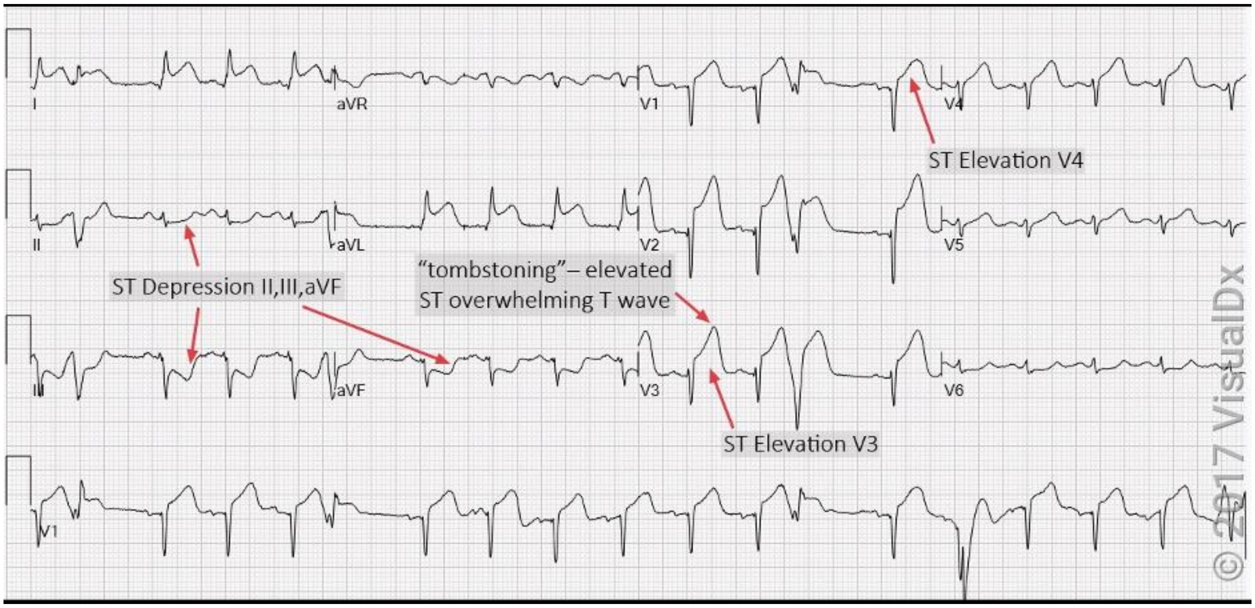

This patient has ST elevation in leads I, aVL, V1, V2, V3, and V4. This meets criteria for an anterolateral STEMI.

STEMI Example #2:

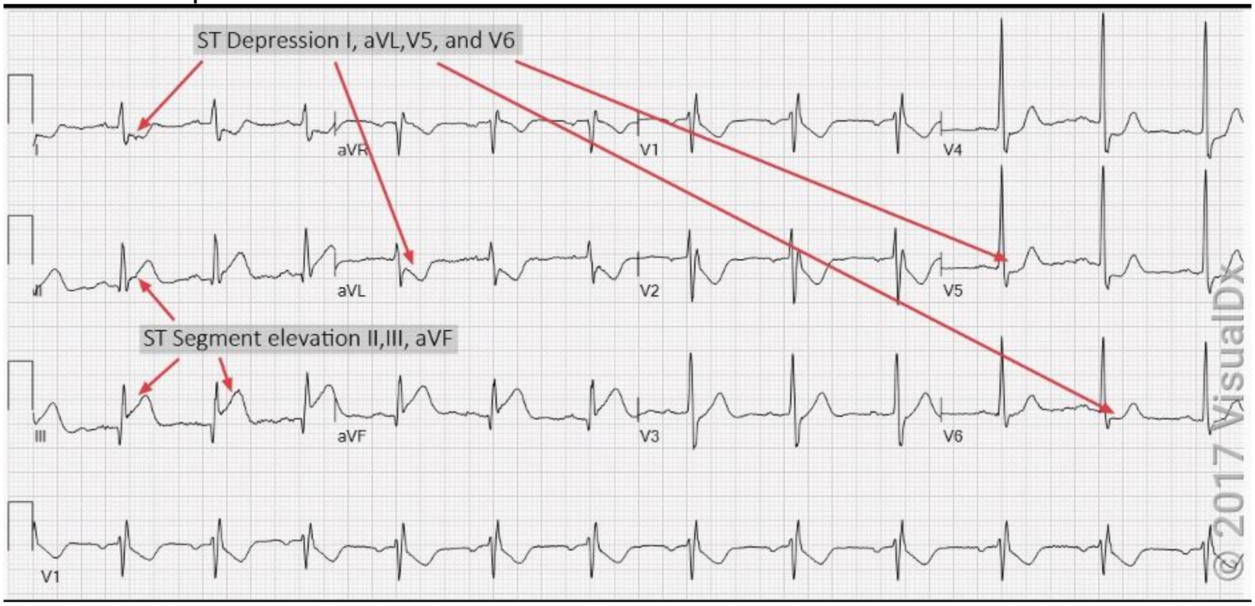

This patient has ST elevation in leads II, III, and aVF. Therefore, this patient meets criteria for an inferior STEMI.

Question 2:
Does this ECG demonstrate a STEMI?

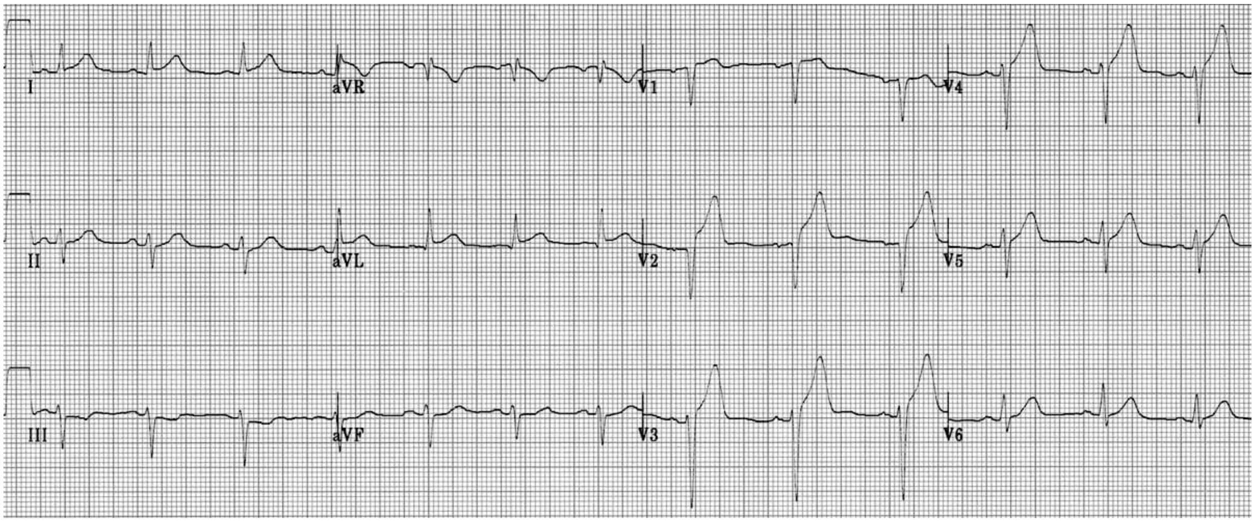

a) No, this ECG does not demonstrate a STEMI
b) Yes, this ECG shows ST elevation in leads I, aVL, and V1-V5; therefore, it is a STEMI

Correct Answer: That is correct! This shows ST elevations ≥1mm in leads I, aVL, and V1-V5. Therefore this is an anterolateral STEMI.

Incorrect Answer: That is incorrect. The correct answer is “b) Yes this a STEMI.” This ECG shows ST elevations ≥1mm in leads I, aVL, and V1-V5. Therefore this is an anterolateral STEMI.

<u>Other ECG findings in ACS</u>

Beyond the ST segment elevations seen in STEMI, there are other ECG findings often seen in ACS. These include: T-wave inversions, ST depressions (as noted on ECGs above), and Q waves. T waves are typically upright; although there are many causes of T wave inversions, myocardial ischemia is one important cause. Therefore, your suspicion for ACS should increase when you see T wave inversion on an ECG. ST segments are typically flat (level with the TP segment). When ST segments are depressed, this is suggestive of myocardial ischemia and should also increase your suspicion of ACS. Q waves are characterized by an initial downward deflection in the QRS complex. There are many causes of Q waves, but one important cause is an old myocardial infarction. When you see Q waves on contiguous leads in an ECG, it should raise your concern that the patient has had a prior myocardial infarction, which should also increase your suspicion for ACS on today’s presentation. Here is an example of an ischemic ECG findings:

Ischemic ECG #1:

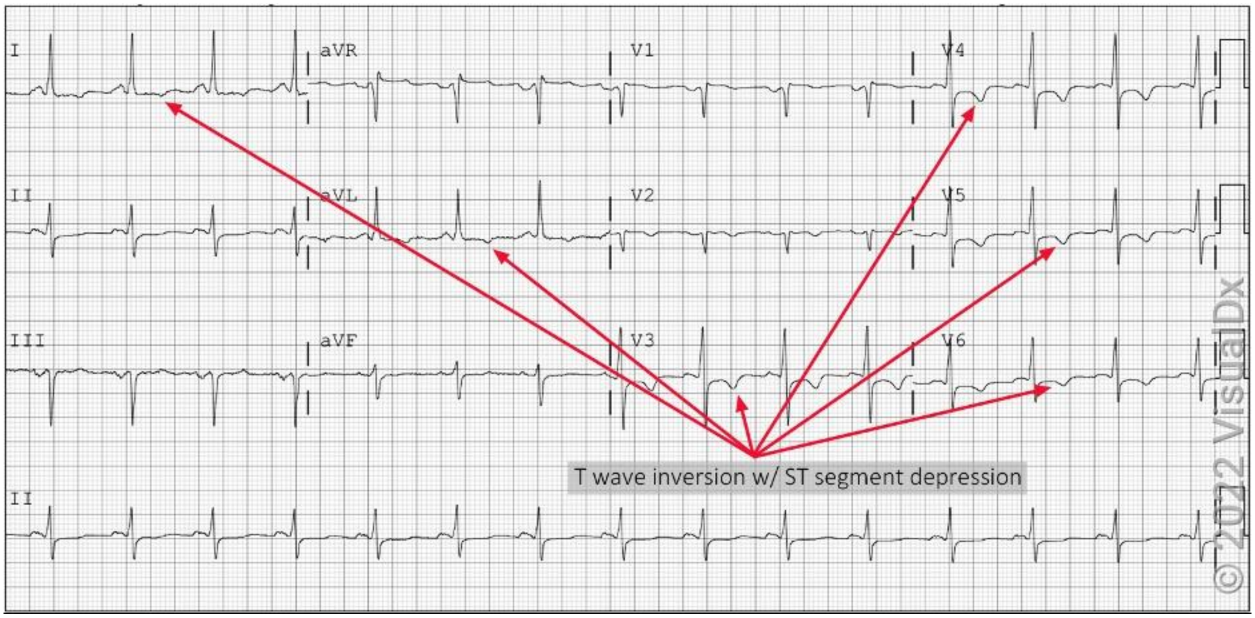

<u>NSTEMI</u>

Patients with NSTEMIs, by definition, will not have an ECG demonstrating a STEMI. Diagnosis of NSTEMI requires blood testing for cardiac biomarkers (ie, troponin). Troponin is a protein found in all muscle cells but diagnostic tests can measure the kind of troponin that is only in cardiomyocytes; the presence of elevated troponins indicates that some cardiomyocytes have died or infarcted. The diagnosis of an acute NSTEMI requires abnormal troponins that are rising or falling; the exact degree to which the troponin level must rise or fall will depend on the specific troponin assay used as your hospital. Therefore, if the initial troponin is abnormal, you should obtain a repeat troponin after approximately 3 hours to determine if it is changing. There are some conditions which can cause chronically elevated troponin levels, such as chronic kidney disease and heart failure. In these conditions, the repeat troponin level should be nearly identical to the initial level. *Importantly, any initial abnormal troponin level is highly suggestive of ACS; therefore patients presenting with possible ACS symptoms and abnormal troponin level should be treated as though they have ACS (ie, given aspirin) until proven otherwise.*

<u>Unstable Angina</u>

Patients with unstable angina will not have an ECG demonstrating a STEMI and will also have a normal (ie, negative) troponin level. Patients with unstable angina will often have ischemic findings on ECG, such as T-wave inversions or ST depressions. Unstable angina is a clinical diagnosis based on history. For example, if a patient requires several weeks of exertional chest pain that is now occurring with only minimal exertion, then you should be highly concerned for unstable angina (if their ECG does not demonstrate a STEMI and their troponin is normal).

<u>Diagnosis:</u>

Because ACS is a life-threatening condition, diagnostic studies should be obtained rapidly in these patients. The key diagnostic studies for ACS are: an ECG, a troponin test, and a focused history and physical exam. Importantly, there are no highly specific or sensitive physical exam findings in ACS. If the initial troponin is abnormal, you should obtain a repeat troponin after approximately 3 hours. However, if the initial troponin is abnormal, you should NOT delay ACS treatment while awaiting the repeat troponin.

Question #3:
A patient presents with chest pain and you are concerned about ACS. What diagnostic studies are most important for evaluating for ACS in the Emergency Department?

a. Complete blood count (CBC), basic metabolic profile (BMP)
b. Chest xray (CXR) and blood gas
c. ECG and troponin
d. Thorough physical exam and urinalysis

Correct Answer: That is correct! ECG and troponin are the key diagnostic tests needed to diagnose STEMI and NSTEMI.

Incorrect Answer: That is incorrect. The correct answer is “c) ECG and troponin.” ECG and troponin are the key diagnostic tests needed to diagnose STEMI and NSTEMI.

Treatment

Treatment of ACS is time-sensitive and should be initiated as soon as possible. Early evidence-based treatment of ACS has been shown to substantially reduce mortality and morbidity. *The initial treatment of ACS is aspirin 300 mg which should be given to any patient for whom you have any suspicion for ACS.* Because a single dose of aspirin has relatively few side effects and reduces mortality in ACS, aspirin should be given in all possible ACS cases unless the patient reports a history of severe allergy to aspirin.

Once the diagnosis of ACS (including unstable angina) has been made, you should proceed with the other key elements of emergency department treatment, which include clopidogrel 600 mg, a heparin bolus of 4000 units iv followed by a heparin drip, and admission to the hospital. If percutaneous coronary intervention or thrombolytics are available at your hospital, these treatments should be initiated from the emergency department for STEMI cases presenting within 24 hours of symptom onset, as per your hospital’s protocols.

Question #4:
A 49 year-old patient present presents with shortness of breath and bilateral leg swelling. Her vital signs are normal. Her ECG demonstrates some T-wave inversions but does not demonstrate a STEMI. After evaluating her, you are most worried about heart failure but are also considering the possibility of ACS, which you think is less likely. The patient has no known medication allergies. You have ordered a troponin test

and an echocardiogram. What treatment should you initiate right now while awaiting these tests?

a) No treatment is necessary right away, since this patient is stable and her diagnosis is still unclear.
b) Furosemide iv should be given immediately, since heart failure is at the top of your differential diagnosis.
c) Aspirin 300 mg should be given immediately, since ACS is on your differential diagnosis and early administration of aspirin reduces mortality in ACS.
d) Supplemental oxygen should be administered immediately.

Correct Answer: That is correct! Since aspirin can be life-saving in ACS, you should order aspirin immediately if you are considering ACS in your differential diagnosis.

Incorrect Answer: That is incorrect. The correct answer is “c) Aspirin 300 mg should be given immediately.” Since aspirin can be life-saving in ACS, you should order aspirin immediately if you are considering ACS in your differential diagnosis.

Question #5:

A 61 year-old male with history of hypertension and chronic kidney disease presents with chest pain and shortness of breath. His vital signs are normal. His initial ECG is normal. His initial troponin is abnormal (positive). You are unsure of how to interpret his abnormal troponin since he has underlying chronic kidney disease. How should you proceed?

a) Administer aspirin 300 mg immediately and obtain a repeat troponin to see if the troponin level is changing.
b) Obtain a repeat troponin, but wait to give aspirin until the repeat troponin results.
c) Discharge home with follow-up for chronic kidney disease.

Correct Answer: That is correct! Any patient with an abnormal troponin should be given aspirin. Ideally, you would give aspirin even before the troponin results if the patient’s symptoms are potentially concerning for ACS. An initial troponin that is abnormally elevated should always be followed by a repeat troponin a few hours later to differentiate a true NSTEMI from other causes of chronic myocardial injury such as renal failure.

Incorrect Answer: That is incorrect. The correct answer is “c) Administer aspirin and obtain a repeat troponin.” Any patient with an abnormal troponin should be given aspirin. Ideally, you would give aspirin even before the troponin results if the patient’s symptoms are potentially concerning for ACS. An initial troponin that is abnormally elevated should always be followed by a repeat troponin a few hours later to differentiate a true NSTEMI from other causes of chronic myocardial injury such as renal failure.

Question #6:

A 42 year-old female with history of diabetes patient presents with jaw pain, diaphoresis, and light-headedness. Her vital signs are normal. Her ECG demonstrates T wave inversion and ST depressions, but does not demonstrate a STEMI. Her initial troponin is abnormal and her repeat troponin is rising significantly. Based on this workup, you conclude the patient has an NSTEMI. What treatment should this patient be given?

a) Aspirin 300 mg
b) Clopidogrel 600 mg
c) Heparin bolus
d) All of the above

Correct Answer: That is correct! Aspirin, clopidogrel, and heparin all reduce morbidity or mortality in STEMIs and NSTEMIs. These treatments should be given immediately in the Emergency Department

Incorrect Answer: That is incorrect. The correct answer is “d) All of the above.” Aspirin, clopidogrel, and heparin all reduce morbidity or mortality in STEMIs and NSTEMIs. These treatments should be given immediately in the Emergency Department

Conclusion:

Thank you for completing this ACS Refresher Training for Emergency Department Providers. **You can save lives by always considering the diagnosis of ACS, ordering ECGs and troponins on patients with possible ACS, and administering early aspirin to patients with possible ACS.**

References:

Amsterdam EA, Wenger NK, Brindis RG, Casey DE Jr, Ganiats TG, Holmes DR Jr, Jaffe AS, Jneid H, Kelly RF, Kontos MC, Levine GN, Liebson PR, Mukherjee D, Peterson ED, Sabatine MS, Smalling RW, Zieman SJ. 2014 AHA/ACC Guideline for the Management of Patients with Non-ST-Elevation Acute Coronary Syndromes: a report of the American College of Cardiology/American Heart Association Task Force on Practice Guidelines. J Am Coll Cardiol. 2014 Dec 23;64(24):e139-e228. doi: 10.1016/j.jacc.2014.09.017. Epub 2014 Sep 23. Erratum in: J Am Coll Cardiol. 2014 Dec 23;64(24):2713-4. Dosage error in article text. PMID: 25260718.

Collet JP, Thiele H, Barbato E, Barthélémy O, Bauersachs J, Bhatt DL, Dendale P, Dorobantu M, Edvardsen T, Folliguet T, Gale CP, Gilard M, Jobs A, Jüni P, Lambrinou E, Lewis BS, Mehilli J, Meliga E, Merkely B, Mueller C, Roffi M, Rutten FH, Sibbing D, Siontis GCM; ESC Scientific Document Group. 2020 ESC Guidelines for the management of acute coronary syndromes in patients presenting without persistent ST-segment elevation. Eur Heart J. 2021 Apr 7;42(14):1289-1367. doi:

10.1093/eurheartj/ehaa575. Erratum in: Eur Heart J. 2021 May 14;42(19):1908. Erratum in: Eur Heart J. 2021 May 14;42(19):1925. Erratum in: Eur Heart J. 2021 May 13;: PMID: 32860058.

Ferry AV, Anand A, Strachan FE, Mooney L, Stewart SD, Marshall L, Chapman AR, Lee KK, Jones S, Orme K, Shah ASV, Mills NL. Presenting Symptoms in Men and Women Diagnosed With Myocardial Infarction Using Sex-Specific Criteria. J Am Heart Assoc. 2019 Sep 3;8(17):e012307. doi: 10.1161/JAHA.119.012307. Epub 2019 Aug 20. PMID: 31431112; PMCID: PMC6755854.

Gulati M, Levy PD, Mukherjee D, Amsterdam E, Bhatt DL, Birtcher KK, Blankstein R, Boyd J, Bullock-Palmer RP, Conejo T, Diercks DB, Gentile F, Greenwood JP, Hess EP, Hollenberg SM, Jaber WA, Jneid H, Joglar JA, Morrow DA, O'Connor RE, Ross MA, Shaw LJ. 2021 AHA/ACC/ASE/CHEST/SAEM/SCCT/SCMR Guideline for the Evaluation and Diagnosis of Chest Pain: A Report of the American College of Cardiology/American Heart Association Joint Committee on Clinical Practice Guidelines. Circulation. 2021 Nov 30;144(22):e368-e454. doi: 10.1161/CIR.0000000000001029. Epub 2021 Oct 28. Erratum in: Circulation. 2021 Nov 30;144(22):e455. PMID: 34709879.

O'Gara PT, Kushner FG, Ascheim DD, Casey DE Jr, Chung MK, de Lemos JA, Ettinger SM, Fang JC, Fesmire FM, Franklin BA, Granger CB, Krumholz HM, Linderbaum JA, Morrow DA, Newby LK, Ornato JP, Ou N, Radford MJ, Tamis-Holland JE, Tommaso CL, Tracy CM, Woo YJ, Zhao DX, Anderson JL, Jacobs AK, Halperin JL, Albert NM, Brindis RG, Creager MA, DeMets D, Guyton RA, Hochman JS, Kovacs RJ, Kushner FG, Ohman EM, Stevenson WG, Yancy CW; American College of Cardiology Foundation/American Heart Association Task Force on Practice Guidelines. 2013 ACCF/AHA guideline for the management of ST-elevation myocardial infarction: a report of the American College of Cardiology Foundation/American Heart Association Task Force on Practice Guidelines. Circulation. 2013 Jan 29;127(4):e362-425. doi: 10.1161/CIR.0b013e3182742cf6. Epub 2012 Dec 17. Erratum in: Circulation. 2013 Dec 24;128(25):e481. PMID: 23247304.

Thygesen K, Alpert JS, Jaffe AS, Chaitman BR, Bax JJ, Morrow DA, White HD; Executive Group on behalf of the Joint European Society of Cardiology (ESC)/American College of Cardiology (ACC)/American Heart Association (AHA)/World Heart Federation (WHF) Task Force for the Universal Definition of Myocardial Infarction. Fourth Universal Definition of Myocardial Infarction (2018). J Am Coll Cardiol. 2018 Oct 30;72(18):2231-2264. doi: 10.1016/j.jacc.2018.08.1038. Epub 2018 Aug 25. PMID: 30153967.

3. Pocket cards

The champions distributed small pocket cards to all ED providers (physicians, clinical officers, and nurses). These pocket cards reviewed the basic of AMI diagnosis and treatment. Providers were encouraged to bring their pocket cards to work with them every day.

The pocket cards contained the following information:

Acute Coronary Syndrome

Symptoms:
- -Chest pain (acute)
- -Shortness of breath/dyspnea
- -Light-headedness
- -Nausea
- -Diaphoresis/sweating
- -Jaw pain
- -Heart failure symptoms

Workup:

12-lead ECG, serial troponins (approx. 3 hrs apart)

Acute MI:

STEMI: ≥1 mm ST elevation in 2 contiguous leads

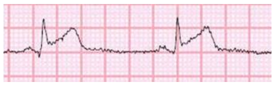

NSTEMI: elevated troponin (rising or falling)

Unstable angina:

ACS symptoms that are worsening or occurring at rest

EMD Treatment:
- -Aspirin 300 mg STAT (*for any suspicion of ACS*)
- -Clopidogrel 600 mg (for acute MI)
- -Heparin 4000 units IV bolus (for acute MI)
- -Consider tenecteplase if STEMI < 24 hours
- -Consider nitrate or morphine for pain control
- -Consider statin

A picture of the pocket card is below:

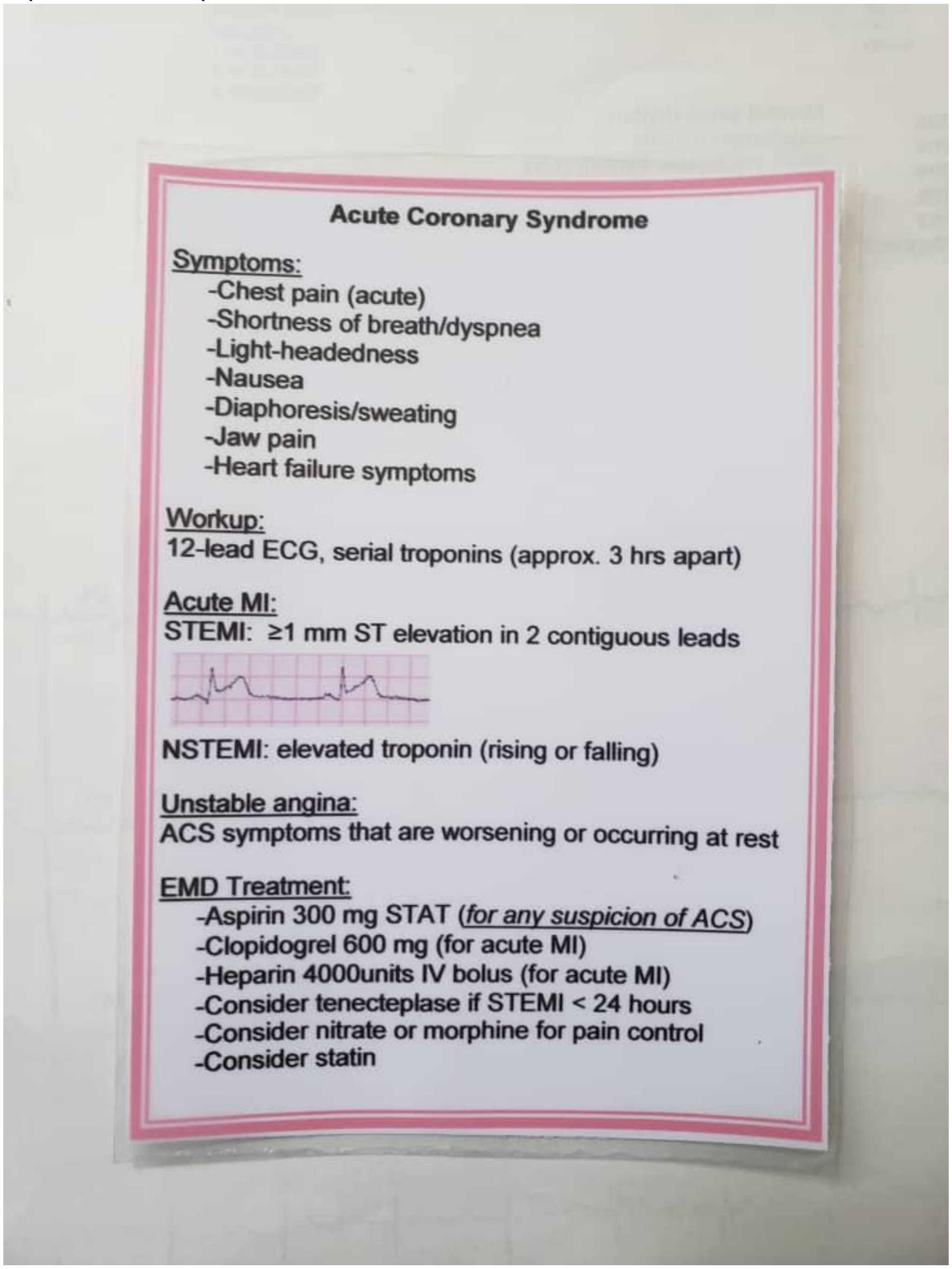

Acute Coronary Syndrome

<u>Symptoms:</u>

- -Chest pain (acute)
- -Shortness of breath/dyspnea
- -Light-headedness
- -Nausea
- -Diaphoresis/sweating
- -Jaw pain
- -Heart failure symptoms

<u>Workup:</u>

12-lead ECG, serial troponins (approx. 3 hrs apart)

<u>Acute MI:</u>

STEMI: ≥1 mm ST elevation in 2 contiguous leads

NSTEMI: elevated troponin (rising or falling)

<u>Unstable angina:</u>

ACS symptoms that are worsening or occurring at rest

<u>EMD Treatment:</u>

- -Aspirin 300 mg STAT (*for any suspicion of ACS*)
- -Clopidogrel 600 mg (for acute MI)
- -Heparin 4000units IV bolus (for acute MI)
- -Consider tenecteplase if STEMI < 24 hours
- -Consider nitrate or morphine for pain control
- -Consider statin

4. Champions

A designed nurse champion and physician champion were selected by the head of the ED. The champions were responsible for encouraging the staff to provide good AMI care and ensuring delivery of the full MIMIC intervention. The champions received a stipend of 100,000 Tanzanian shillings (approximately 40 USD) per month for their efforts. Detailed champion responsibilities are listed below.

Nurse Champion Responsibilities:

a. Encourage staff to provide good AMI care
b. Ensure delivery of the full MIMIC intervention
c. Ensure that triage nurses are using the special red triage cards appropriately
d. Distribute the link to the online training module to all ED nurses, including new and rotating nurses
e. Ensure all nurses complete the online training module
f. Distribute the pocket cards to all ED nurses
g. On a daily basis, ensure that there is an adequate supply of aspirin in the ED medicine cabinet
h. Recognize nurses who provide exceptional AMI care with a congratulatory certificate
i. Follow-up on cases where nurses fail to provide appropriate AMI care

Physician Champion Responsibilities:

a. Encourage staff to provide good AMI care
b. Ensure delivery of the full MIMIC intervention
c. Distribute the link to the online training module to all ED physicians, including new and rotating physicians
d. Ensure all physicians complete the online training module
e. Distribute the pocket cards to all ED physicians
f. Recognize physicians who provide exceptional AMI care with a congratulatory certificate
g. Follow-up on cases where physicians fail to provide appropriate AMI care
h. Ensure that physicians are distributing the patient educational pamphlets to patients with AMI

5. Patient Educational Pamphlets

Patient educational pamphlets are stored in the ED and given to patients diagnosed with AMI. The physician who diagnoses the patient with AMI is primarily responsible for giving the pamphlet to them. Nurses may also distribute pamphlets as they see fit. In the event that the patient is too ill to receive the pamphlet, the pamphlet is given to a family member who is with them. In addition to the physical pamphlets, a digital version of the pamphlet is displayed on television screens in the waiting room. These screens display rotating educational messages, including the AMI educational pamphlet.

The final version of the pamphlet was in Swahili:

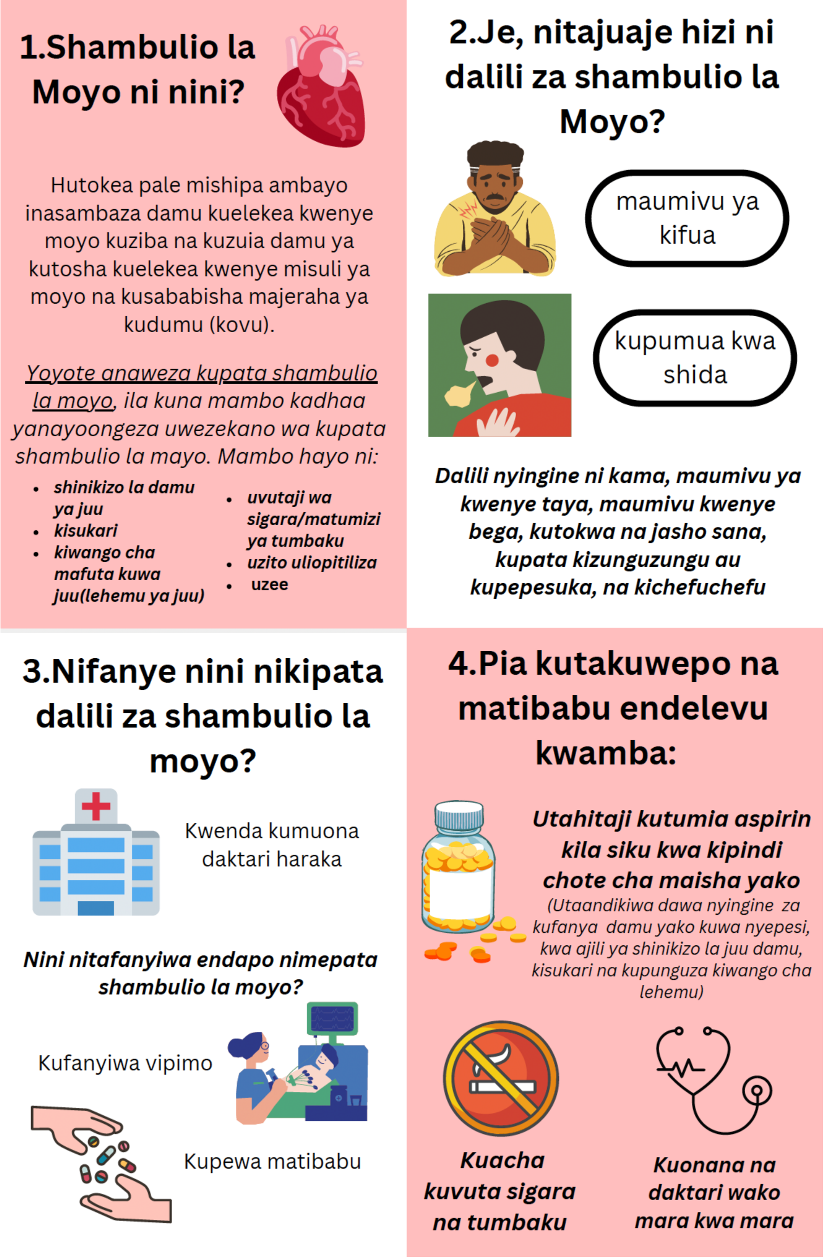

An English version of the pamphlet is below:

What is a Heart Attack?

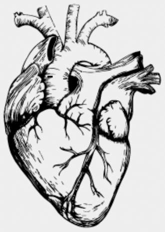

Happens when a blockage prevents enough blood supply to your heart, and might leave some permanent damage to your heart

__Anyone can get a heart attack__, but some risk factors include:

- ***High blood pressure***
- ***Diabetes***
- ***High cholesterol***
- ***Smoking***
- ***Obesity***
- **Older individuals**

Recognizing a Heart Attack

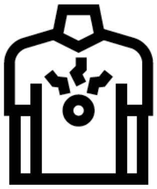

Chest pain

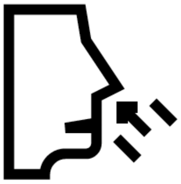

Shortness of Breath

Other symptoms may include: ***jaw pain, arm pain, sweating, light-headness,*** *and* ***nausea***

What happens if you have a heart attack?

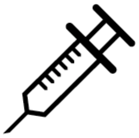

Undergo medical tests

Admitted to the hospital

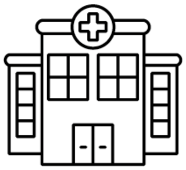

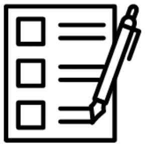

Take medications

See a specialist for your heart condition

Continuing care

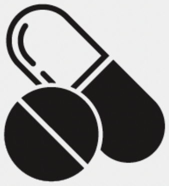

Take Aspirin for the rest of your life
(you may be prescribed other medications to thin your blood, for hypertension, diabetes and high cholesterol as well)

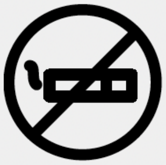

Stop Cigarettes & Tobacco

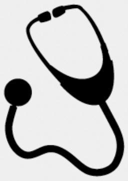

Regular check-ups with your doctor

digital version of the pamphlet was converted into four different slides displayed on the television screens in the waiting room:

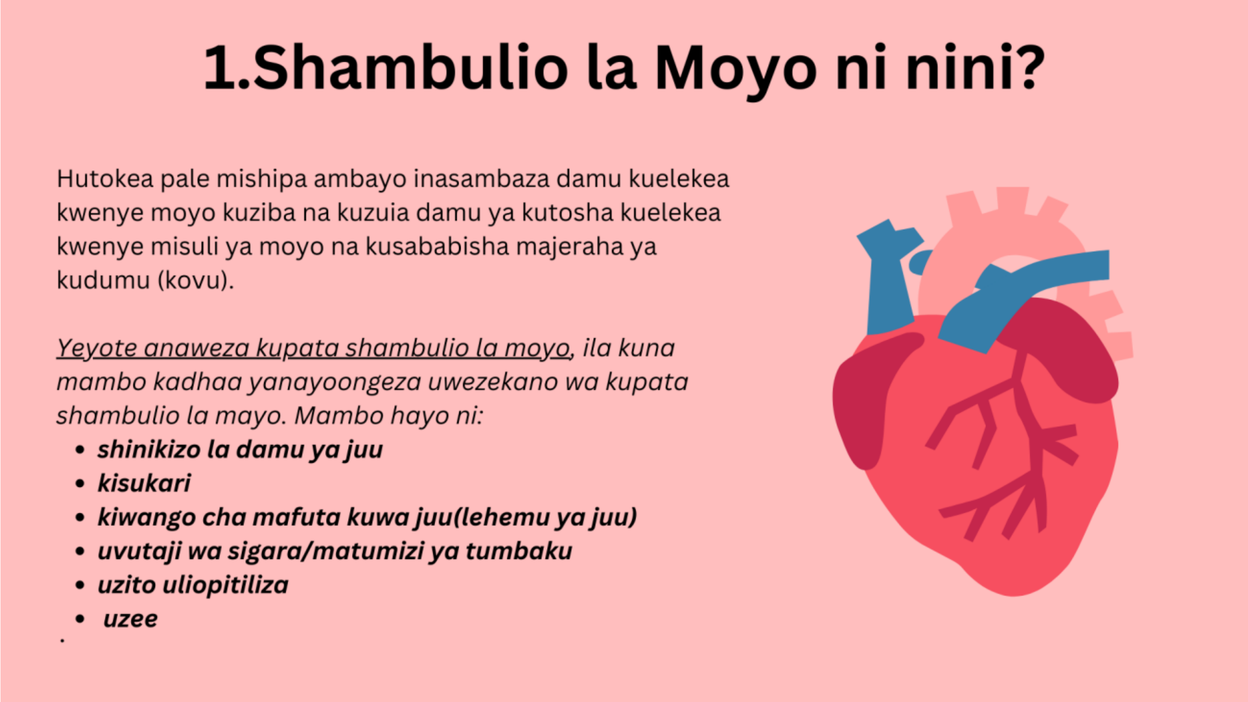

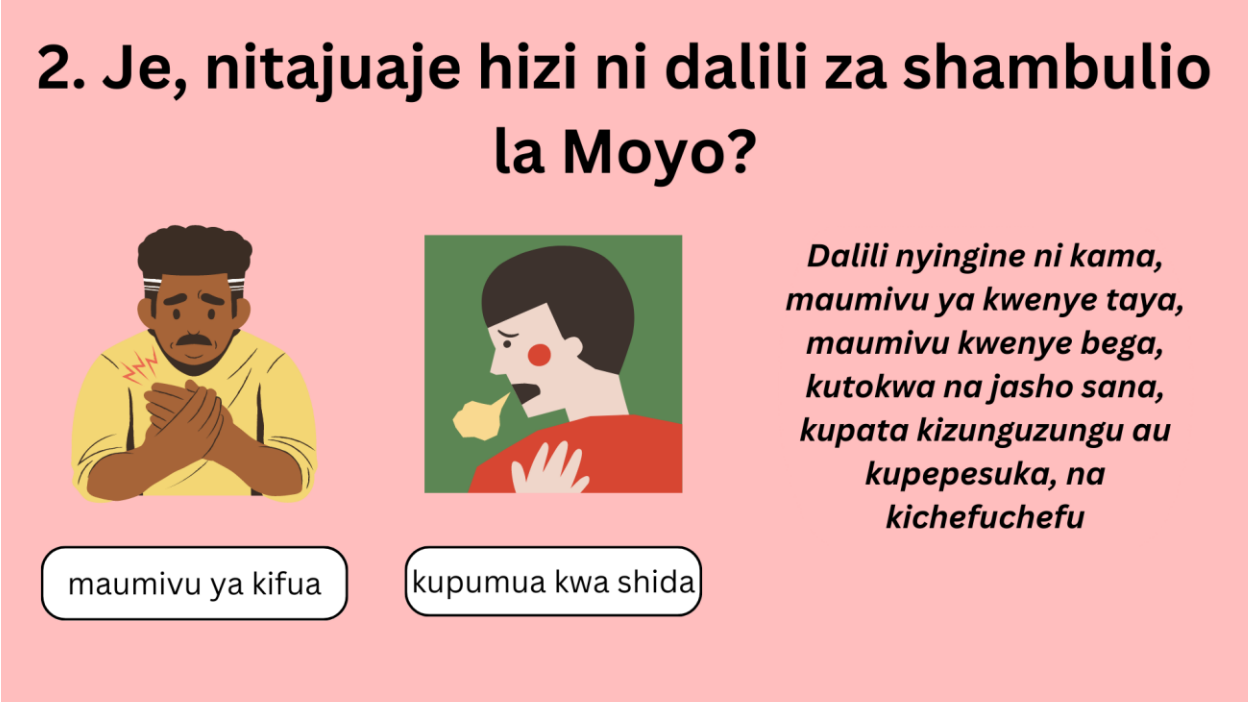

3.Nifanye nini nikipata dalili za shambulio la moyo?

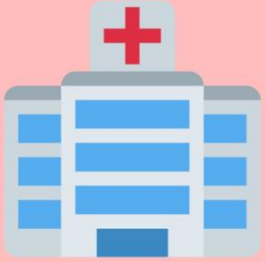

Nenda kamwone daktari haraka

Nini nitafanyiwa endapo nimepata shambulio la moyo?

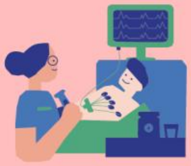

Kufanyiwa vipimo

Kupewa matibabu

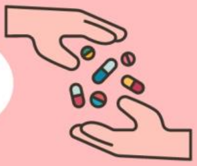

4.Pia kutakuwepo na matibabu endelevu kama:

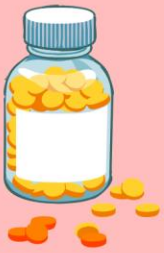

Utahitaji kutumia aspirin kila siku kwa kipindi chote cha maisha yako

(Utaandikiwa dawa nyingine za kufanya damu yako kuwa nyepesi, kwa ajili ya shinikizo la juu damu, kisukari na kupunguza kiwango cha lehemu)

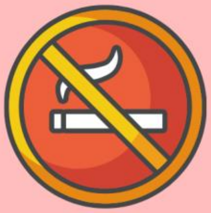

Kuacha kuvuta sigara na tumbaku

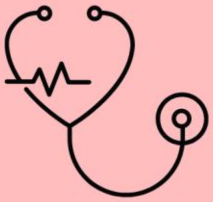

Kuonana na daktari wako mara kwa mara
